# Differentiated trust: navigating credibility landscapes in community-engaged research

**DOI:** 10.3389/fpubh.2026.1846231

**Published:** 2026-05-18

**Authors:** Steven L. Arxer

**Affiliations:** Department of Sociology & Anthropology, The University of Texas at Arlington, Arlington, TX, United States

**Keywords:** community knowledge, community-engaged research, differentiated trust, participatory partnerships, researcher positionality, trust work

## Abstract

Community-engaged research (CEnR) depends on mutual credibility between researchers and communities, yet the trust environment in which these collaborations operate has shifted as public confidence becomes more conditional and politically structured. Drawing on scholarship in the sociology of trust, epistemic injustice, and related fields, this paper applies the concept of differentiated trust to community-engaged research. Differentiated trust describes a pattern in which people grant credibility selectively across knowledge authorities based on institutional experience, political identity, and information environment. Differentiated trust asks a related question about how credibility has already been sorted among competing authorities before the collaboration begins. The paper examines how differentiated trust illuminates the role of epistemic trust, epistemic injustice, and identity work in contexts where communities are navigating competing authorities on the health topics research addresses. The paper argues that attending to differentiated trust can sharpen the practical relevance of these concepts for CEnR design and evaluation.

## 1 Introduction

Public trust in scientific institutions in the United States has undergone a reconfiguration. Overall confidence in scientists recovered to 76% in 2024, near its pre-pandemic level, but the share of adults reporting a great deal of confidence declined from 39% in April 2020 to 26% in October 2024 (1). A substantial gap has opened along partisan lines, with 88% of Democrats but only 66% of Republicans expressing at least a fair amount of confidence (1). This divergence signals a longer trajectory accelerating since 2018, driven by shifts on both ends of the political spectrum (2), and it operates internationally, with trust varying substantially by political orientation, education, and prior engagement with scientific institutions across 68 countries (3). What has emerged is not a wholesale loss of confidence but a reconfiguration, in which the criteria by which scientific authority is evaluated have become more varied and more politically structured (4, 5).

Community-engaged research (CEnR) operates within this shifting landscape. CEnR is a research orientation encompassing a range of approaches united by a commitment to equitable collaboration between academic and community partners (6–8). CEnR depends on mutual credibility, and community engagement has been shown to strengthen research relevance, build local capacity, and in some cases improve health outcomes (9–12). The CEnR literature has developed substantial scholarship on trust, including conceptual models in which trust cuts across multiple domains of the collaborative process (13, 14), empirical work demonstrating that trust develops across stages of collaboration and varies along dimensions such as vulnerability, shared values, and power sharing (15–17), and sustained attention to how power asymmetries and researcher positionality shape whose knowledge carries authority (18–20).

Trust has been understood as varying by context. Whether trust is granted depends on who is being trusted, what they are being trusted about, and what experience the person granting trust has had with them (21–23). Credibility is also shaped by power. Whose knowledge counts, and on what basis, varies by social position (24–26). This points to a pattern that can be called differentiated trust, in which trust is not absent but granted to some knowledge authorities and withheld from others, based on how people have experienced institutions, how political identity shapes their evaluations, and what information sources they encounter. Multidimensional trust illuminates how trust operates among collaborators along multiple dimensions within partnerships (15, 16). Differentiated trust asks a related question about what happens outside the partnership, how credibility has already been sorted among competing authorities before the collaboration begins. Differentiated trust has implications for how CEnR understands the credibility landscape in which it operates. To examine those implications, this paper draws on three concepts from CEnR and adjacent literatures, epistemic trust (27), epistemic injustice (25), and identity work (28, 29), each of which addresses how knowledge, credibility, and power operate in research partnerships. When communities have already sorted the credibility of competing health authorities before a collaboration begins, these concepts help clarify what researchers are navigating and what existing approaches need to account for. Specifically, they open questions about how communities decide which health authorities to trust, how those decisions have been shaped by the way institutions have treated them, and how the researcher’s own credibility is read against that background. CEnR’s frameworks and practices are well positioned to respond to differentiated trust, but doing so requires attending to how credibility operates beyond the partnership itself.

## 2 Differentiated trust and the contemporary terrain

Survey data from 2024 through 2026 illustrate how trust in the health domain varies by source. A national survey of 2,716 parents conducted in the summer of 2025 found substantial variation in whom respondents trusted for health information (30). Respondents who expressed skepticism toward the Centers for Disease Control (CDC) simultaneously reported trusting their pediatrician at 81% (30). Wellness influencers, despite their prominence in public discourse, were trusted by only 14% of respondents who identified as supporters of the Make America Healthy Again (MAHA) movement, and 84% of respondents overall attributed financial motivations to influencers (31). Over 80% of respondents across party lines supported stronger regulation of food additives and processed foods (30). Concerns about chronic disease, pharmaceutical industry practices, and food regulation are broadly shared, even as respondents differ in which authorities they trust to address them (32, 33). Nationally, 17% of parents reported delaying or skipping vaccines in early 2025, up from 10% in 2023 (34), and by early 2026, overall trust in the CDC to provide reliable vaccine information had declined to 47% (35). The information environment has been recognized as a health outcome in its own right, as trust in healthcare providers has been identified as the strongest predictor of vaccine uptake, with reliance on alternative information sources associated with lower uptake (36, 37).

These patterns illustrate differentiated trust. Rather than reflecting a simple decline in confidence, they reveal a reconfiguration of how credibility is allocated. Credibility is socially constructed and shaped by institutional experience, social position, and the kind of knowledge in question (23, 25, 26). While some individuals reject scientific authority broadly, the survey data suggest that most make judgments about specific sources based on their own experience with those sources and with the institutions those sources represent (1, 23). In the health domain, this means that trust is granted selectively. This selectivity signals evaluations not only of competence but of whether the institutions producing health knowledge are serving community interests. Concerns about corporate influence on research, pharmaceutical industry conduct, food safety, and environmental exposure are real questions about whose interests health knowledge serves. How people sort credibility across these competing authorities reflects how institutions, including research institutions, have treated them and are perceived to treat them.

A community’s history with institutions shapes community research before research inquiry begins (38). Research with indigenous communities has documented how prior experiences with extractive research and community evaluations of institutional authority shape whether and how external knowledge is received (39, 40). Trust building depends on understanding these prior relationships and the community’s existing ways of evaluating credibility (39, 41). Communities of color during the COVID-19 pandemic experienced a “continuum of trauma” across historical, cultural, and social dimensions that shaped how institutional health communications were received (42), drawing on generations of extractive research and institutional betrayal (40, 43). Analysis of community-based participatory research contexts has documented how power dynamics rooted in these histories shape what kinds of knowledge are valued and whose expertise is recognized (20).

The contemporary landscape extends these dynamics. Trust judgments are now shaped not only by histories of institutional harm but by a contested information environment in which political figures, wellness influencers, healthcare providers, and federal agencies compete as health authorities. Recent community-based participatory research confirms the field’s capacity to operate in these conditions, including vaccine hesitancy interventions with Hispanic and Black communities (44), work addressing anti-Black racism and institutional mistrust among African, Caribbean, and Black Canadians (45), and a scoping review finding that community participation enhanced credibility and uptake across 23 vaccination promotion studies (46). Community-based participatory research has been described as “uniquely equipped” to address the current trust landscape (47).

Trust operates within partnerships and cuts across multiple domains of the collaborative process (15, 16). Differentiated trust extends that work by attending to what precedes the collaboration. By the time a researcher partners with a community, people have often decided which authorities they find credible on the health topics the research addresses and which they do not. Those prior judgments, shaped by experience with physicians, public health agencies, political figures, and online health voices, are part of what the partnership inherits.

## 3 Trust work through differentiated trust

Trust work refers to the ongoing processes through which researchers and communities negotiate and sustain mutual credibility as contributors to shared knowledge production. The term draws on its use in adjacent literatures (48–50) while specifying it for CEnR contexts in which trust varies across the authorities that people in those communities encounter. Three concepts, each already present within or adjacent to CEnR, address successive dimensions of this challenge. The first, epistemic trust, concerns how communities orient to competing knowledge sources. The second, epistemic injustice, concerns how institutional power shapes which orientations are treated as credible. The third, identity work, concerns how the researcher’s own positioning within this landscape affects the collaboration. Together they trace a path from how credibility is distributed, to how that distribution is produced, to how researchers navigate it in practice. These are not exhaustive, and other concepts, including work on epistemic oppression (24) and epistemic resistance (26), may prove equally productive.

### 3.1 Epistemic orientations and the landscape of competing authorities

Research on epistemic trust has documented that trust varies depending on the knowledge source and on the recipient’s relational history with that source (27, 51, 52). Responses range from openness when the source is perceived as reliable, to guarded closure when prior experience has involved unreliable or harmful communication, to uncritical acceptance when the source is familiar or perceived as aligned with the recipient’s concerns. These responses are context-dependent and selective, meaning that the same person may be receptive to health information from one source and skeptical of it from another.

The practical implication for CEnR is that what communities bring to a collaboration is not only a history with research institutions but a set of active credibility relationships with other authorities who address the same health topics the research addresses. These relationships structure how the researcher’s knowledge is received, what counts as a credible claim, and whose framing of a health concern is taken seriously. Parents, for example, may trust their pediatrician while expressing skepticism toward the CDC and evaluating political figures and wellness influencers as health information sources, all while holding real concerns about food safety and chronic disease that are broadly shared across party lines (30).

An analogy from education is instructive here. In the tradition of differentiated instruction, the point is not simply that learners differ but that the differences say something about what knowledge means to different learners and whether what is being taught is organized around their interests and experience (53). Analogously, how people distribute trust is not simply a matter of varied preferences for information sources. It reflects people’s evaluations of whether the people and organizations responsible for health knowledge are responsive to their concerns. Over 80% of parents across party lines support stronger food safety standards (30), yet they disagree sharply on which authorities to trust. This suggests that the issue is less about the concerns themselves and more about whose interests the institutional response is perceived to serve. CEnR’s commitment to recognizing community knowledge as legitimate and sharing power in knowledge production (6, 8) is relevant here, and recent frameworks such as the BEE model’s emphasis on foregrounding community-identified priorities (54) move in this direction.

### 3.2 Institutional credibility and the production of trust orientations

Credibility is socially distributed and shaped by power. Testimonial injustice occurs when a person’s credibility is systematically deflated due to identity-based prejudice, and hermeneutical injustice occurs when shared vocabulary is insufficient for articulating significant dimensions of experience (25). These dynamics have been documented within participatory research, where power asymmetries can marginalize community knowledge even within structures designed to be equitable (18), and in communities’ relationships with institutions more broadly, where accumulated experiences of research that extracted from communities without returning benefit, racialized medical experimentation, and broken institutional commitments shape how institutional communications are evaluated (40, 42, 43).

These institutional histories shape how people grant credibility in the present. Concerns about how chronic illness is managed, how pharmaceutical companies operate, and what is in the food supply are grounded in direct experience of a healthcare system that many communities perceive as inadequate to their needs (32, 33). When institutional responses frame these concerns primarily as misinformation, they diminish the standing of those who are drawing on lived experience to raise legitimate questions about how health systems serve them (25).

For CEnR, this means that the ways people in a community evaluate credibility have been shaped by how institutions, including research institutions, have treated them as knowers. A researcher who engages with community concerns seriously, treating the experiential knowledge behind them as legitimate even when specific claims are scientifically contested, undertakes trust work consistent with core CEnR principles (6, 8). Community members are evaluating whether the research enterprise and its funding structures are accountable to them. Research on power dynamics within CEnR has documented how these patterns shape whose knowledge is valued in collaborative settings (18, 20).

### 3.3 Researcher identity and competing credibility

Research on identity in community-based participatory research has established that how researchers and community members perceive each other’s roles, expertise, and institutional location affects how the collaboration unfolds (19, 28, 29). Shared racial and ethnic identity between researchers and community members has been found to generate “proxy trust” that facilitates entry, though professional training continues to shape what knowledge carries authority in practice (19). Multi-site research has documented how the gap between how researchers understand their own role and how community members experience it creates tensions that require active management (19, 20).

Differentiated trust adds a layer to this dynamic. The researcher’s institutional affiliation, funding sources, and disciplinary background signal alignment with or distance from the authorities that community members trust or distrust. In communities where trust varies, the same researcher may be received very differently by different people in the community. Researcher identity shapes the collaboration in practice (19), and trust building requires ongoing relational investment (55, 56). The positionality the researcher negotiates now also includes positioning relative to authorities whose credibility is shaped by political identity and institutional experience. Community health workers, faith leaders, and peer specialists have been identified as trusted mediators who bridge institutional and community ways of knowing (48, 56), and identifying who occupies these roles and how they are trusted by different segments of the community is a relevant part of research design.

## 4 Discussion

The CEnR trust literature has developed nuanced, contextually grounded approaches to understanding trust within collaborative research (14, 15, 17, 19). Differentiated trust complements this work. It draws attention to how community members have already allocated credibility across different sources of health information, and how those judgments shape what happens in the research. Four implications for practice follow.

First, several CEnR approaches for understanding community context speak directly to this challenge. Community engagement studios (57) were designed to surface community priorities before research design begins. Community engagement studios (57) were designed to surface community priorities before research design begins. River of life exercises (41) map the historical relationships that shape how communities relate to institutions. Both focus on the community’s history with research. These tools could also be used to explore the community’s current relationships with the range of authorities that shape how health knowledge is evaluated, from pediatricians and public health agencies to political leaders and online health voices. Mapping these relationships is not only about understanding barriers but about identifying where trust already operates and how CEnR can build from it. The Engage for Equity measures (14, 58) assess trust within partnerships. It may also matter to understand trust outside the partnership, since how people in the community relate to outside authorities shapes what happens inside the collaboration. The BEE Research Framework (54) does this by centering what communities themselves identify as pressing. In this context, those priorities may include concerns about pharmaceutical accountability, food regulation, or environmental exposure that are shared across political lines (30).

Second, how researchers engage with community health concerns matters for the credibility of the collaboration. Research on epistemic injustice in participatory contexts has shown that community knowledge can be set aside even in formally participatory settings (18). Community members raise concerns that come from living within the healthcare system (32, 33). Treating those concerns as misinformation rather than engaging with the experience behind them diminishes the standing of the person raising them (25). The point is not whether the researcher agrees with every claim. It is whether the researcher takes the experience behind the concern seriously. CEnR has long held that community knowledge is legitimate and that power in interpretation should be shared (6, 8). Under these conditions, that principle has specific practical consequences. A researcher who dismisses concerns that over 80% of parents across party lines share (30) risks undermining the credibility of the collaboration itself.

Third, researcher positionality requires ongoing engagement in the current environment. Research on identity in CEnR has established that how the researcher is perceived affects the collaboration (19), and multi-site analysis has shown that researcher self-perception and community experience of the researcher often diverge in ways that create real tensions (20). When trust is differentiated along political lines, the researcher’s institutional affiliation and funding sources mean different things to different community members. Some may see a university-affiliated researcher as credible. Others may see that same affiliation as a mark of the institutions they have come to doubt. This positioning cannot be resolved once and set aside. It requires ongoing relational work and committement (55, 56). Working with community members already trusted across institutional and community lines, such as community health workers and faith leaders (48, 56), may be especially valuable when the researcher’s own credibility varies across the community.

Fourth, what collaborations leave behind matters. Trust typologies developed from partnership data have modeled trust as a developmental process that moves through stages, from initial skepticism to mutual reliance and shared authority over what the research means (17). In this environment, the later stages of that process matter more because the community’s relationship to the researcher is only one of many credibility relationships the community is managing at any given time. A collaboration that ends by strengthening the community’s ability to assess health claims across multiple authorities based on the merits of those claims leaves a different legacy than one that simply builds trust in the research institution. CEnR has long been committed to capacity building (6, 9), and differentiated trust gives that commitment a specific direction today. The goal is not to replace one authority with another but to support the community’s independent capacity to evaluate competing claims.

The distribution of credibility across the range of authorities people encounter reflects a fundamental question about whether the research enterprise is accountable to the communities it studies, one that ultimately determines the legitimacy of the collaboration. Communities are asking whether the institutions that fund, design, and publish health research serve them. Research that strengthens people’s capacity to evaluate competing health claims independently and to participate in shaping what counts as relevant knowledge engages with differentiated trust at this level.

The concepts examined here are illustrative rather than exhaustive. Work on epistemic oppression has identified forms of exclusion that go beyond testimonial and hermeneutical injustice, including the silencing that occurs when speakers anticipate that their testimony will not be received (24). Similarly, epistemic resistance describes how marginalized knowers develop independent knowledge practices in response to oppressive conditions (26). Both offer resources for understanding differentiated trust. The conditions described here are drawn from U.S. data and may not generalize directly to other contexts, though trust differentiation appears to operate internationally (3). Future research should examine how trust, institutional histories, and political identity interact within specific CEnR contexts, and should test how existing approaches and practices perform under conditions of differentiated trust.

## 5 Conclusion

CEnR operates in a trust landscape that has changed substantially. Trust in scientific institutions has become more conditional and politically divided, and competing information authorities shape how health claims are evaluated. CEnR attends to context and history in partnership building. What differentiated trust adds is the recognition that trust has not disappeared but shifted, and that identifying where credibility currently resides for a given community offers CEnR a starting point for engagement rather than a barrier to it.

Differentiated trust reframes the challenge. Rather than asking how to build trust where it is absent, it asks how credibility is currently distributed and what that distribution means for how a collaboration enters a community (23, 25, 26). The three concepts examined here, epistemic trust, epistemic injustice, and identity work, offer ways of reading that distribution, understanding how it was produced, and navigating it in practice. At its core, this is about whether health research serves the communities it is meant to benefit, a question central to CEnR’s commitments. Paying attention to how trust is differentiated rather than simply present or absent positions CEnR to engage with the credibility landscape as it actually exists rather than the one its models have traditionally assumed.

## Data Availability

The original contributions presented in the study are included in the article/supplementary material, further inquiries can be directed to the corresponding author/s.
